# Advances of autoimmune rheumatic diseases related to malignant tumors

**DOI:** 10.1007/s00011-023-01780-6

**Published:** 2023-09-28

**Authors:** Miaomiao Zhao, Liangyu Mi, Yuli Ji, Xiaoyao He, Yanan Gao, Yuting Hu, Ke Xu

**Affiliations:** 1 Third Hospital of Shanxi Medical University, Shanxi Bethune Hospital, Shanxi Academy of Medical Sciences, Tongji Shanxi Hospital, Taiyuan, 030032 China; 2 Department of Statistics, School of Public Health, Shanxi Medical University, Taiyuan, China; 3 Shanxi Bethune Hospital, Shanxi Academy of Medical Sciences, Tongji Shanxi Hospital, Third Hospital of Shanxi Medical University, Taiyuan, 030032 China

**Keywords:** Malignancies, Autoimmune rheumatic diseases, Paraneoplastic rheumatic syndromes, Tumor therapy, Immune-related adverse reactions

## Abstract

**Background:**

Malignant neoplasms are a well-recognized global public health concern, with significant impacts on human health and quality of life. The interplay between tumors and autoimmune rheumatic diseases is complex, and the resulting tumor-associated rheumatic diseases represent a rare and intricate group of conditions that occur in the context of malignant tumors. In addition, various rheumatic diseases can arise as a consequence of oncology treatment. These diseases present with intricate clinical manifestations and pathological features, often rendering them challenging to diagnose and impacting patients' quality of life. Despite this, they have yet to be fully recognized.

**Methods:**

This article presents a literature review of published original articles and review articles concerning paraneoplastic rheumatic syndromes and rheumatic diseases associated with cancer treatment. We conducted a comprehensive literature search in PubMed, Web of Science and Google Scholar databases, excluding duplicated and irrelevant studies. In cases of duplicated research, we selected articles with higher impact factors for the review.

**Results:**

This review focuses on the clinical features, diagnosis, and treatment of paraneoplastic rheumatic diseases, as well as the pathogenesis of these diseases. Additionally, we summarize the autoimmune rheumatic diseases associated with cancer treatment. Ultimately, the goal of this review is to enhance recognition and improve the management of autoimmune rheumatic diseases related to tumors.

## Introduction

Malignant tumors represent the second largest public health issue worldwide due to their aggressive nature and high mortality rate. According to incomplete statistics, 7–10% of tumor patients may exhibit clinical manifestations of paraneoplastic syndromes, which are more commonly associated with rheumatic diseases, second only to endocrine and neurological disorders. In 2006, Andras et al. [1] first introduced the concept of “paraneoplastic rheumatic syndrome”, which denotes a paraneoplastic syndrome predominantly characterized by rheumatoid-like symptoms. Tumor tissues can release various humoral factors containing tumor antigens, and foreign tumor antigens can be recognized by the human immune system, eliciting the production of corresponding antibodies or T cells targeting normal tissues, thereby affecting distant tissues such as bone and muscle. Chronic inflammation is a key factor in the development of both tumors and autoimmune rheumatic diseases. During tumorigenesis, oncogenes are expressed in target organ cells, inducing the release of chemotactic factors that recruit and activate immune cells such as natural killer (NK) cells, natural killer T (NKT) cells, macrophages, dendritic cells (DCs), and neutrophils [2, 3]. The factors secreted by these cells possess the ability to induce either the survival or apoptosis of tumor cells. The persistent infiltration of innate immune cells and the resulting inflammatory response precipitates the infiltration of lymphocytes, localized antigen presentation, and subsequent activation of antigen receptors. Activation of lymphocytes by either self or novel antigens stimulates the release of cytokines, including interferon-γ (IFN-γ), leading to organ toxicity and functional impairment, ultimately resulting in corresponding symptoms of rheumatic diseases [4].

The treatment of tumors is intricately intertwined with rheumatic pathologies. Conventional modalities for tumor management encompass surgical intervention, radiation therapy, and chemotherapy. Radiation and chemotherapy may elicit abnormal immune responses, leading to autoimmune disorders in which the immune system attacks the body's own tissues. Additionally, tissue damage and inflammatory reactions induced by radiation and chemotherapy can trigger self-reactive immune responses, ultimately resulting in the onset of rheumatic diseases. With the advancements in molecular biology and oncology, tumor immunotherapy has gradually gained widespread application in the clinical setting for various types of cancer, including breast cancer, melanoma, lymphoma, and non-small cell lung cancer, among others, exhibiting favorable therapeutic efficacy. While tumor immunotherapy provides benefits to patients, it may also disrupt the balance of the body's self-immune tolerance, leading to immune-related adverse events. Rheumatic-like adverse reactions may occur when the therapy induces muscle, bone, or joint lesions.

In clinical practice, some patients with paraneoplastic rheumatic syndromes are highly suspected of having tumors, but pathological evidence of tumors cannot be found. Therefore, the number of patients with paraneoplastic syndromes presenting as rheumatic diseases is far lower than the actual level. Moreover, the rheumatic manifestations caused by tumor treatment pose a significant challenge to the management of cancer patients. Thus, early recognition of tumors and rheumatic diseases is particularly crucial for patient diagnosis and prognosis.

## Paraneoplastic rheumatic syndromes

Paraneoplastic syndrome (PNS) refers to the pathological conditions that occur in remote locations such as the cardiovascular, endocrine, neuromuscular, hematologic, gastrointestinal, and cutaneous systems, caused by abnormal immune reactions including cross-immunity, autoimmunity, immune complex deposition, or other unknown reasons, rather than the direct infiltration, compression, or metastasis of corresponding tissue or organs by tumors. In most cases, PNS appears prior to the onset of tumors and evolves in parallel with the progression of the primary lesion. Paraneoplastic rheumatic syndromes (PRSs) are characterized by prominent rheumatic symptoms, including paraneoplastic polyarthritis, palmar fasciitis and polyarthritis syndrome, remitting seronegative symmetrical synovitis with pitting oedema, tumor-associated myositis, hypertrophic osteoarthropathy, pancreatic panniculitis with polyarthritis, polymyalgia rheumatica and tumor-induced osteomalacia. Rheumatoid-like manifestations may precede, occur simultaneously with, or occur subsequent to other symptoms of malignancy.

### Tumor-induced osteomalacia

Tumor-induced osteomalacia (TIO) is a metabolic bone disease characterized by increased renal phosphate excretion and is the most common form of acquired hypophosphatemic osteomalacia. Clinically, it is manifested by progressive bone pain, muscle weakness, and in severe cases, limb and height shortening, pathological fractures, and skeletal deformities. TIO tends to occur more frequently in individuals aged 30–60, and there is no significant difference in its incidence between males and females.

Tumors that induce TIO typically exhibit a slow-growing pattern, and can be found in various parts of the human body, with a higher incidence in the limbs, followed by the head, neck, and maxillofacial regions. Among the 895 patients diagnosed with TIO, 97.9% of the tumors were benign and relatively small in size [5]. The most commonly observed tumor type was the phosphaturic mesenchymal tumor with mixed connective tissue subtype (PMT), followed by osteosarcoma. In addition, patients with malignant tumors such as prostate cancer, breast cancer, small cell lung cancer, and colorectal cancer may also experience symptoms of TIO [6]. Laboratory examination of TIO patients reveals a decrease in blood phosphate levels, an increase in urine phosphate levels, and a decrease or normal level of 1,25-dihydroxyvitamin D3. In most cases of TIO, the elevated levels of fibroblast growth factor 23 (FGF-23) and/or Frizzle-4 protein produced by the tumor are responsible for the symptoms [7]. Both RNA and protein levels of FGF-23 are overexpressed in PMT [8]. FGF-23 binds to receptors on the basolateral membrane of renal tubular epithelial cells, suppressing the expression of sodium-dependent phosphate transport protein 2a (NaPi-2a) and sodium-dependent phosphate transport protein 2c (NaPi-2c) in the kidney, resulting in increased urinary phosphate excretion. Simultaneously, FGF-23 suppresses the activity of renal 1α-hydroxylase and promotes the activity of 24-hydroxylase, leading to insufficient active vitamin D and reduced intestinal phosphate absorption, further exacerbating hypophosphatemia. In addition, upregulation of hypoxia-inducible factor 1α (HIF-1α) and fusion genes FN1-FGFR1 and FN1-FGF1 can also induce overexpression, leading to abnormal phosphate metabolism and skeletal mineralization disorders in patients. Apart from FGF-23, matrix extracellular phosphoglycoprotein (MEPE), FGF-7 and secreted frizzled-related protein 4 (sFRP4) can inhibit phosphate transport in the extracellular matrix. Among these, only sFRP4, like FGF-23, can also suppress the activity of renal 1α-hydroxylase, leading to the occurrence of hypophosphatemia [9, 10].

The tumors that cause TIO are mostly occult and difficult to detect. Once a definitive diagnosis is made, tumor localization is crucial. ^18^F-fluorodeoxyglucose (^18^F-FDG), PET-CT, octreotide imaging, MRI, as well as the combination of B-mode ultrasound and CT are effective methods for localizing TIO tumors [11]. Following tumor resection, TIO symptoms can be relieved, and blood FGF-23 levels can be restored to normal. For those who cannot undergo surgery or have incomplete tumor resection, replacement therapy with phosphates and active vitamin D may be an option, while FGF-23 monoclonal antibodies may be a potential targeted therapeutic agent.

### Palmar fasciitis and polyarthritis syndrome

Palmar fasciitis and polyarthritis syndrome (PFPAS) is a rare syndrome characterized by progressive polyarthritis, tenosynovitis, palmar fasciitis, and bilateral finger end contractures. It presents as acute, symmetrical, diffuse joint pain and swelling of mild severity, accompanied by bilateral hand flexion contractures, which can progress to Dupuytren's contracture, typically involving the wrist, metacarpophalangeal joints, and proximal interphalangeal joints [12]. Laboratory tests for PFPAS show normal levels of inflammation markers, rheumatoid factor, anti-nuclear antibodies, and anti-neutrophil cytoplasmic antibodies. However, immunoglobulins and complement deposition can be seen in the palmar fascia, and elevated tumor markers such as CA125 or CA19-9 are often present, providing clues for diagnosis [13, 14]. Joint X-rays generally exhibit non-specific manifestations. Ultrasound imaging may reveal hypoechoic nodules in the palmar fascia, while MRI can detect changes in the T1 and T2 weighted images of the corresponding fascia and subcutaneous tissue in fibromatosis [15].

PFPAS is frequently linked with neoplasms, with a prevalence rate of 36.8% in female patients diagnosed with ovarian gland cancer. Additionally, PFPAS can manifest in other types of malignancies, including but not limited to lung cancer, prostate cancer, breast cancer, and gastrointestinal tumors [13]. After chemotherapy in patients with advanced lung adenocarcinoma, there is an improvement in pulmonary radiographic manifestations, disappearance of arthritic symptoms, and a decline in the tumor marker carcinoembryonic antigen (CEA), further supporting the notion that PFPAS is a paraneoplastic disorder [16]. After undergoing tumor radical surgery for ovarian cancer, patients experience relief of synovitis symptoms, however, joint contracture persists. In addition, the hand contracture symptoms worsen with tumor recurrence [17, 18]. Moreover, PFPAS symptoms have also been observed in some patients with endometrial cysts and paraovarian cysts. PFPAS manifestations often indicate advanced tumor progression, and therefore, the overall prognosis of PFPAS patients is poor. Treatment with non-steroidal anti-inflammatory drugs (NSAIDs), glucocorticoids, and immunosuppressive agents has not shown significant efficacy.

The pathogenesis of PFPAS is currently unclear, with the main histological features being fibroblast proliferation and increased extracellular matrix components [19]. In some patients, significantly elevated levels of connective tissue growth factor have been found in serum, which may be a potential pathogenic mechanism. Ovarian cancer releases various cytokines, such as vascular endothelial growth factor (VEGF), insulin-like growth factor (IGF), and transforming growth factor-β (TGF-β). Additionally, the expression of TGF-β, IGF, and VEGF has been found in lung cancer, and TGF-β has been associated with the metastasis of lung cancer. Based on the association between PFPAS and ovarian and lung cancer, these cytokines are believed to play an important role in the progression of the disease.

### Hypertrophic osteoarthropathy

Hypertrophic osteoarthropathy (HOA) is a disease characterized by abnormal proliferation of skin, soft tissue, and periosteum of the limbs. It is more commonly observed in males and is typically manifested as tibial and femoral pain, adjacent joint pain or synovitis, and clubbing of the fingers [20]. HOA can be categorized into two distinct subtypes: primary hypertrophic osteoarthropathy (PHO) and secondary hypertrophic osteoarthropathy (SHO).

PHO is a rare genetic disorder that primarily affects the skin, bones, and soft tissues. PHO can be inherited in two ways: autosomal recessive and incomplete autosomal dominant. The main causative genes for PHO include the HPGD gene, which encodes 15-hydroxyprostaglandin dehydrogenase (15-PGDH), and the SLCO2A1 gene, which encodes prostaglandin transporter (PGT) [21, 22]. Mutations in HPGD and SLCO2A1 result in increased levels of prostaglandin E2 (PGE2) in vivo. PGE2 is known to induce vasodilation, promote local tissue proliferation, and stimulate osteoblasts and osteoclasts, ultimately leading to clinical manifestations such as clubbing, hyperhidrosis, periosteal proliferation, and acro-osteolysis [23, 24]. Prostaglandin E2 (PGE2) is a common immunomodulatory factor that plays a crucial role in maintaining immune homeostasis in the body. However, elevated serum levels of PGE2 can severely disrupt immune balance and lead to increased proliferation and migration of tumor cells. Therefore, it is imperative for PHO patients to be vigilant about the occurrence of gastrointestinal tumors and undergo screening gastroscopy and colonoscopy examinations.

SHO is a condition that frequently arises as a secondary manifestation of various underlying disorders, including tumors, pulmonary infections, inflammatory diseases, and chronic hypoxia [25]. A majority of cases (90%) of SHO are linked to diverse malignancies, encompassing bronchogenic carcinoma, solitary fibrous tumor of the pleura or mesothelioma, thymoma, atrial myxoma, gastrointestinal malignancies, renal cell carcinoma, osteosarcoma, nasopharyngeal carcinoma, and hematological malignancies [25]. It is noteworthy that non-small cell lung cancer is the most commonly associated malignancy [26]. Platelet-derived growth factor (PDGF) produced by tumor cells is closely associated with the development of heterotopic ossification (HO). Released from small blood vessels, PDGF can increase vascular distribution and permeability, promote the growth of mesenchymal cells, and subsequently enhance new bone formation and formation of heterotopic bone [27]. Moreover, vascular endothelial growth factor (VEGF) can promote the proliferation and differentiation of osteoblasts [28]. Elevated levels of VEGF have been detected in the serum of patients with tumors, while VEGF levels tend to return to normal after tumor resection. The diagnosis of SHO primarily relies on imaging studies. X-ray findings typically include periosteal ossification and osteophyte formation, while bone scans show increased radioactive uptake in newly formed bone tissue [25]. Therefore, in cases where patients present with symptoms or signs of HOA, and X-rays reveal symmetric osteophyte formation in the distal long bones without associated bone destruction or fracture, caution should be exercised for the possibility of SHO.

### Remitting seronegative symmetrical synovitis with pitting oedema

Remitting seronegative symmetrical synovitis with pitting oedema (RS3PE) is more commonly seen in the elderly and is characterized by symmetrical involvement of small joints (such as hand and wrist joints, and flexor tendon sheaths) accompanied by pitting edema on the dorsum of the hands and feet. It is associated with various rheumatic diseases, such as rheumatoid polymyalgia, giant cell arteritis, polymyositis, systemic sclerosis, and late-onset peripheral spondyloarthritis. RF is negative and the prognosis is relatively good. Currently, it is believed that the disease represents an early manifestation of certain malignancies. Among the 331 RS3PE patients, 11.18% had concurrent genitourinary and gastrointestinal tumors, followed by lung cancer and hematological malignancies [29]. It is noteworthy that RS3PE can be induced by oral hypoglycemic agents, such as dipeptidyl peptidase-4 inhibitors, diuretics, benzodiazepines, and insulin therapy [29]. The aforementioned drugs can induce VEGF-A mRNA and protein expression, resulting in increased levels of VEGF in serum and the development of RS3PE [30]. The etiology and pathogenesis of RS3PE remain unclear, but VEGF is considered a potential pathogenic mechanism.

When the following symptoms occur in a patient, RS3PE should be suspected: (1) acute onset; (2) symmetrical punctate concave edema on the hands and feet; (3) onset age ≥ 60 years; (4) good response to steroids; (4) negative serum RF and ACPA; (5) no radiographic joint erosion [31]. In terms of treatment, RS3PE responds well to glucocorticoid therapy, but RS3PE combined with tumors has a poor response to glucocorticoid therapy. When elderly patients present with the above symptoms, accompanied by systemic symptoms (such as unexplained fever, weight loss), negative serum antibodies, and no significant bone erosion changes on imaging, particularly when the response to low-dose glucocorticoid therapy is poor, potential tumors should be considered.

### Paraneoplastic polyarthritis

Paraneoplastic polyarthritis (PP) is a type of arthritis associated with cancer, but often without direct evidence of tumor invasion or metastasis, nor specific histological or radiological features. PP typically affects elderly patients and has a rapid onset. While PP may present in various ways, it can resemble rheumatoid arthritis or migratory arthritis [32, 33]. A more common manifestation of PP is a serum-negative asymmetric arthritis, with involvement of the lower extremities being more frequent and the involvement of the small joints of the hands being less common. Synovitis in PP patients usually presents with acute asymmetric (91%), polyarticular (34%), oligoarticular (48%), or monoarticular (18%) onset, with a mean age of onset at 50 years, and a significantly increased male ratio compared to RA [34]. Laboratory examinations may reveal a significant increase in inflammatory markers such as erythrocyte sedimentation rate (ESR) and C-reactive protein (CRP) in patients with PP. Furthermore, 23% of PP patients test positive for rheumatoid factor (RF) and 11% for anti-cyclic citrullinated peptide (CCP) antibodies, which adds to the diagnostic challenge of the disease [34].

At present, the pathogenesis of PP remains unclear. In patients with renal cell carcinoma and concurrent oligoarthritis, clonal T-cell receptor gene rearrangement can be detected in both tumor tissue and synovial tissue, which may suggest that PP involves a cross-reactive immune response between tumor cells and synovial tissue with shared antigens [35]. At present, the pathogenesis of PP remains unclear. In patients with renal cell carcinoma and concurrent oligoarthritis, clonal T-cell receptor gene rearrangement can be detected in both tumor tissue and synovial tissue, which may suggest that PP involves a cross-reactive immune response between tumor cells and synovial tissue with shared antigens [36]. At least some cases of PP, particularly those with anti-CCP positivity, may be associated with an immune reaction induced by guanidinated proteins, a tumor antigen. PP patients have poor responses to treatment with NSAIDs, glucocorticoids, and immunosuppressants. Successful treatment of malignancy can improve arthritic symptoms, but the recurrence of the tumor does not necessarily result in the reappearance of arthritis [37, 38]. Therefore, when the clinical presentation involves atypical joint involvement and a poor response to conventional anti-rheumatic treatments such as glucocorticoids, the presence of malignancy should be considered.

### Cancer-associated myositis

Cancer-associated myositis (CAM) is typically defined as the development of cancer within 3 years after the diagnosis of dermatomyositis [39], and it has been associated with various malignant tumors, including lung cancer, breast cancer, pancreatic cancer, ovarian cancer, cervical cancer, gastrointestinal cancer, and hematological malignancies [40]. The majority of cancers are diagnosed either concurrently with or within one year of dermatomyositis diagnosis. Although the risk of cancer gradually decreases within three to five years, the risk remains higher compared to the general population [41–43]. Inflammatory myopathies have a close association with neoplasms, with dermatomyositis (DM) being the most commonly observed subtype. Polymyositis (PM) can also be associated with neoplasms, albeit to a lesser extent than DM. The incidence rate of neoplasms in DM patients ranges from 3.8% to 7.7%, while the risk of neoplasms in PM patients is relatively lower compared to DM [44]. Elderly patients with DM who experience rapid progression, dysphagia, skin necrosis, leukocytoclastic vasculitis, and positive anti-p155 or anti-nuclear matrix protein-2 (NXP-2) antibodies are at a higher risk of developing neoplasms [45, 46]. Laboratory examination shows that the serum anti-p155 antibody levels are elevated in CAM patients. The sensitivity and specificity of anti-p155 antibodies in diagnosing CAM are 78% and 89%, respectively [47]. CAM lacks specific imaging features and is difficult to differentiate from idiopathic inflammatory myopathies.

CAM is an autoimmune disorder, wherein cancer-associated antibodies against transcriptional intermediary factor1-gamma (TIF-1γ) and NXP-2 may induce cross-immune reactions within the body, leading to skin and vascular damage, ultimately resulting in inflammatory reactions in the muscle tissue [48]. 8–21% of adult DM patients test positive for anti-TIF-1γ antibodies, among which 38–80% of patients develop malignant neoplasms at the same time as or after DM diagnosis [49–51]. Anti-TIF-1γ antibodies can deactivate Smad4 ubiquitination, blocking the TGF-β/Smad signaling pathway, leading to TGF-β resistance in cell proliferation and promoting tumor development. In addition, by inhibiting the epithelial-mesenchymal transition, anti-TIF-1γ antibodies induce tumor suppression genes to age, leading to tumor metastasis [52, 53]. Furthermore, 29% of DM patients test positive for anti-NXP-2 antibodies, with 37.5% of anti-NXP-2 positive patients developing tumors within three years of myositis diagnosis [54].

Patients with CAM have a poorer prognosis compared to those with ordinary myositis, with a significant decrease in 5-year survival rate and poor response to treatment with steroids and immunosuppressive agents. Although 53% of patients experience relief after surgery, radiotherapy or chemotherapy, the sustained immune reaction induced by tumors may lead to relapse of myositis [45]. Given the temporal relationship between myositis and tumor occurrence, basic cancer screening should be performed at the time of diagnosis, and tumor monitoring should be conducted annually for the following 3 years.

### Pancreatic panniculitis with polyarthritis

Pancreatic panniculitis with polyarthritis (PPP) is a rare complication of pancreatic disease that is more prevalent in middle-aged men, with approximately half of all patients experiencing no gastrointestinal symptoms. Lipodystrophy tends to occur in the lower extremities, while arthritis commonly affects joints such as the ankle, knee, wrist, and foot [55]. Skin involvement manifests as nodular erythema and nodular vasculitis. PPP is commonly associated with acute or chronic pancreatitis, and approximately 12% of cases are related to pancreatic malignancies, such as pancreatic neuroendocrine tumors, pseudopancreatic cancer, and acinar cell carcinoma [55]. Histological examination can reveal areas of fat necrosis and characteristic adipocytes [56]. Imaging studies reveal intraosseous fat necrosis, which manifests as multiple poorly defined or permeative lytic lesions in the distal extremities. In the advanced stages of the disease, pathological fractures, increased bone density, bone collapse, and joint collapse can occur [57]. On bone scintigraphy, the affected joints exhibit increased uptake, while bone marrow fat necrosis can present as diffuse or heterogeneous uptake [58]. On MR imaging, subcutaneous edema, synovitis, inflammation of the soft tissues around the peripheral joints, bone marrow edema, and bone marrow adipose tissue necrosis can be visualized [59].

Lipase plays a crucial role in the pathophysiological processes of PPP. Lipolysis/fat necrosis and secondary inflammation are not only observed in subcutaneous fat (panniculitis), but also affect fat in other parts of the body. Periarticular fat breakdown and the release of free fatty acids into the joint space may lead to synovitis. Elevated levels of pancreatic enzymes (including lipase and amylase) and lipids have been detected in the synovial fluid of affected joints [60]. Intramedullary fat necrosis can be complicated by joint and skin involvement. Although serum amylase and lipase levels do not reflect the severity of joint disease, sustained high levels of pancreatic enzymes are positively correlated with fat necrosis [57].

In most cases, the treatment of underlying pancreatic disease can improve the symptoms of PPP, but arthritis may persist even after complete remission [60]. The diagnosis of PPP typically relies on skin biopsy, and routine abdominal CT and joint imaging should be performed when PPP is suspected. Most patients with PPP have a poor response to NSAIDs [55, 57].

### Polymyalgia rheumatica

Polymyalgia rheumatica (PMR) is a common inflammatory rheumatic disease in the elderly, characterized by severe pain and stiffness in the shoulders, proximal arms, neck, and bilateral hips. The lifetime risk is 2.4% for females and 1.7% for males, and the incidence increases with age, peaking at age 80 [61, 62]. PMR is closely associated with giant cell arteritis (GCA), with approximately 40–50% of GCA patients showing PMR-like symptoms, and around 20% of PMR patients simultaneously developing GCA [63]. The pathogenesis of the disease is not yet fully understood and may be related to various factors such as genetics, infection, immunity, and inflammation. During the acute phase of the disease, inflammatory markers such as erythrocyte sedimentation rate and C-reactive protein are elevated, and the patient responds well to glucocorticoid therapy [64]. PET/CT imaging can reveal characteristic “Y”-shaped scan images distributed along the inter-spinous bursa, and strong concentration of FDG accumulation in the medial aspect of the shoulder joint extending to the scapular region, lateral aspect of the hip joint, and medial aspect of the knee joint [65]. MRI examination can help detect synovitis associated with PMR [63]. Paraneoplastic PMR is usually associated with bone marrow disorders [66]. When PMR patients are younger than 50 years old, the typical sites affected are limited or asymmetrical, the erythrocyte sedimentation rate is less than 40 mm/h or greater than 100 mm/h, and there is a poor response to low-dose corticosteroids, potential malignancy should be considered [67].

### Paraneoplastic vasculitis

In addition, paraneoplastic vasculitis accounts for approximately 2–5% of all vasculitides, with leukocytoclastic vasculitis being the most common type that primarily involves small vessels of the skin and presents clinically as palpable purpura [68, 69]. More than half of the cases of paraneoplastic vasculitis are secondary to hematologic malignancies such as leukemia, lymphoma, and myelodysplastic syndromes [70]. They can also be observed in urologic, gastrointestinal, and pulmonary cancers [71]. The diagnosis of paraneoplastic vasculitis relies mainly on a detailed medical history and physical examination, and its prognosis is associated with the underlying malignancy. Treatment of vasculitis in clinical practice often involves the use of corticosteroids or combination immunosuppressive agents.

#### Other manifestations

The paraneoplastic lupus-like syndrome, characterized by serositis, Raynaud's phenomenon, and positive ANA antibodies, is relatively rare in patients with malignancies, with only a few cases reported. Recurrent polychondritis is associated with myelodysplastic syndromes and lymphoma [72].

## Rheumatic diseases associated with tumor therapy

### Radiotherapy related rheumatism

Radiation therapy is a treatment method that uses high-energy radiation to kill cancer cells. The process is simple, versatile, and effective, but while it can eliminate tumor cells, it may also damage surrounding normal tissue cells, leading to adverse effects such as depression, nausea and vomiting, decreased immune function, and local skin reactions. Radiotherapy for head and neck cancers may induce damage to adjacent normal salivary and lacrimal glands, leading to dry mouth and dry eye symptoms in patients, which may resemble primary Sjögren's syndrome (pSS). Patients receiving head and neck radiotherapy and pSS patients exhibit upregulation of histone H1.4 and neutrophil collagenase in their saliva, which may be related to post-radiotherapy dry mouth symptoms [73].

Radiotherapy may also give rise to new fibrotic syndromes, such as localized scleroderma. The mechanism of radiation-induced scleroderma is not fully understood, and may be attributed to immune dysregulation in a genetically susceptible background. Radiotherapy can stimulate the expression of IL-4, IL-5, and TGF-β, leading to the activation of fibroblasts and increased synthesis of collagen protein [74, 75]. TGF-β induces the excessive transformation of CD34 + fibroblast progenitor cells into myofibroblasts, resulting in the thickening and hardening of connective tissue. TGF-β can also stimulate its own synthesis, further exacerbating the thickening of connective tissue [76]. In patients with concurrent scleroderma and breast cancer receiving radiotherapy, skin thickening at the site of radiation occurs in as many as 50% of cases [77]. However, due to inadequate sample size, there is currently insufficient evidence to suggest that radiotherapy can lead to localized or systemic skin thickening.

### Chemotherapy related rheumatism

Chemotherapy, as a systemic treatment modality, primarily works by suppressing the growth and proliferation of tumor cells and promoting tumor cell apoptosis. Despite its broad therapeutic scope, the accumulation of drugs may lead to toxic side effects such as gastrointestinal reactions and bone marrow suppression. Various chemotherapy drugs are associated with scleroderma-like diseases, Raynaud's phenomenon, and/or severe digital ischemia, including bleomycin, gemcitabine, carboplatin, and paclitaxel [78–80], as described in Table1.

**Table 1:** Musculoskeletal symptoms associated with tumor chemotherapy drugs

Compound	Side-effects
Bleomycine	Raynaud's syndrome [81] Skin thinning [82]
Gemcitabine	Scleroderma-like lesion [82]
Paclitaxel/docetaxel	SSc-like disease [83]
Vinblastine/vincristine	Raynaud's syndrome [84]
Cisplatin	Raynaud's syndrome Arthralgia, myalgia [85]
Aromatase inhibitors	Osteoporosis [86] Arthralgia, myalgia [87]
5-fluorouracil	Arthralgia, myalgia [88]
Cyclophosphamide	Arthralgia, myalgia [89]
Methotrexate (high-dose)	Arthralgia, myalgia [90]
Tamoxifen	Arthralgia, myalgia [91]

Bleomycin can increase the synthesis of type I collagen and promote the expression of α-smooth muscle actin (α-SMA) and fibronectin, leading to the development of scleroderma in mice [81]. The scleroderma-like lesions induced by bleomycin differ from typical scleroderma, with a predominance in males and lower risk of Raynaud's phenomenon and organ involvement as well as lower positive rates of autoantibodies. When the total dose exceeds 165 mg, the risk of fibrotic changes significantly increases [80, 92].

Paclitaxel and docetaxel can lead to an increase in serum IL-6 levels [93], which can mediate endothelial cell activation and apoptosis in patients with systemic sclerosis (SSc) and participate in the early stages of SSc development [94]. The scleroderma-like lesions induced by paclitaxel and docetaxel can be divided into three stages: (1) edematous stage; (2) edematous and sclerotic stage; and (3) sclerotic stage. In most cases, the disease begins with lower limb edema, which progresses to edematous scleroderma with pigmentation. Histologically, it resembles SSc, with collagen fiber proliferation in all layers of the dermis and fibrosis extending into the subcutaneous adipose tissue [83].

Scleroderma-like lesions induced by gemcitabine often occur in the lower limbs, accompanied by pigmentation. Histologically, there is fibrosis in both the dermis and the fat septa, and there is mild to moderate infiltration of inflammatory cells (lymphocytes) around the blood vessels [82].

Aromatase inhibitors (AIs) have been the primary treatment for postmenopausal women with estrogen receptor-positive breast cancer since their introduction into clinical practice, significantly improving patient survival rates. However, the estrogen depletion caused by AIs can result in adverse effects such as hot flashes, insomnia, slightly increased risk of ischemic heart disease, accelerated bone loss leading to increased risk of osteoporosis, and joint pain. Approximately 50% of patients experience joint pain or worsening pain after receiving AI treatment, and 20% of patients terminate treatment due to joint pain. Joint pain typically improves after a two-week period of drug discontinuation. Premature discontinuation of AIs is the leading cause of AI-induced joint pain.

The bone and joint pain and swelling caused by AIs significantly affect patients' quality of life and medication compliance. The pathogenesis of this condition is not yet clear, and related risk factors include menopause of less than 5 years, a history of taxane chemotherapy, obesity, and a history of arthritis or osteoporosis. Certain single-nucleotide polymorphisms (SNPs) are associated with the musculoskeletal symptoms induced by AIs. Osteoprotegerin (OPG) has a bone-protective effect by inhibiting downstream signaling of RANKL in osteoclasts, and a decrease in OPG levels has been observed in patients with acute rheumatoid arthritis. The SNP (OPG RS2073618) encodes a missense mutation allele and is associated with an increased risk of joint and muscle pain after AI treatment [87], as well as a positive correlation with an increased risk of postmenopausal osteoporosis and fractures [86]. Blocking the estrogen-dependent SNP RS11849538 located in TCL1A leads to increased NF-kB transcription, which can mediate joint inflammation [95]. Genomic analysis has revealed that the intragenic variant RS79048288 within CCDC148 and the intergenic variant RS912571 upstream of PPP1R14C are associated with the occurrence of musculoskeletal symptoms and discontinuation of drug therapy during AIs treatment [96]. Furthermore, the negative correlation between systemic inflammatory cytokines, pain sensitivity, and estrogen levels in patients suggests that estrogen depletion caused by AIs may exacerbate joint inflammation symptoms [97, 98]. There is no standard treatment regimen for musculoskeletal symptoms caused by AIs. Patients may improve their symptoms through regular exercise, vitamin supplementation, analgesics and corticosteroid therapy, acupuncture, and other methods.

The rheumatic manifestations and severity of chemotherapy-induced diseases vary, and whether to discontinue the drug depends on the patient's condition. In some cases, discontinuation of certain drugs does not alleviate the rheumatic symptoms in some patients. If the lesion affects the joints, it can cause joint flexion contracture, which severely affects daily life. Therefore, when the lesion may lead to functional impairment, in addition to discontinuing the drug, oral corticosteroids should also be considered for treatment.

### Rheumatic adverse reactions associated with immune checkpoint inhibitors

In recent years, tumor immunotherapy has become another novel method of cancer treatment following traditional surgery, chemotherapy, radiation therapy, and targeted therapy. It can actively or passively stimulate and regulate the immune system of cancer patients, thereby obtaining specific or non-specific anti-tumor immunity and achieving the goal of controlling malignant tumors. Immune checkpoint inhibitors are a milestone breakthrough in tumor immunotherapy.

Immune checkpoint inhibitors (ICIs) are a class of monoclonal antibodies targeting regulatory immune checkpoint molecules that inhibit T cell activation. By blocking the co-inhibitory signaling pathway, ICIs enhance T cell-mediated anti-tumor immunity and promote immune-mediated clearance of tumor cells. Currently, clinically approved ICIs mainly target cytotoxic T lymphocyte-associated antigen 4 (CTLA-4), programmed cell death protein-1 (PD-1), and programmed cell death ligand-1 (PD-L1). ICIs can enhance non-specific immune reactions in normal tissues/organs of cancer patients, leading to immune-related adverse events (IrAEs) [99]. IrAEs can affect multiple systems, including respiratory, digestive, endocrine, and musculoskeletal. Previous clinical trial data has shown that 54–76% of patients experience irAEs of varying degrees, with rates of up to 90% observed in patients receiving combination immunotherapy [100, 101]. Rh-irAEs, which primarily manifest as musculoskeletal involvement, encompass a spectrum of conditions including arthralgia and inflammatory arthritis, rheumatoid myalgia, myositis, Sjogren's syndrome, and vasculitis. Symptoms are often atypical, with 18.4% of patients treated with ICIs experiencing Rh-irAEs, representing a 1.3-fold increased risk compared to patients who did not receive ICI therapy [102]. The reported incidence of Rh-irAEs varies widely in different studies (1.5–22%), and some cancer patients may have pre-existing musculoskeletal-related symptoms, making it extremely challenging to distinguish these types of irAEs [103].

#### Joint pain and inflammatory arthritis

Joint pain is the most common clinical manifestation of Rh-irAEs, with an incidence rate of 42.1%. It typically occurs around three months after drug use, and the incidence rate is higher in combination immunotherapy than in monotherapy. Inflammatory arthritis (IA) is an important component disease [104]. ICIs-IA, which is characterized by joint involvement, usually affects the upper limb joints, including the shoulder, elbow, wrist, metacarpophalangeal, and proximal interphalangeal joints. The most commonly affected joint in the lower limbs is the knee joint, followed by the ankle and tarsometatarsal joints. Approximately two-thirds of IA patients present with rheumatoid arthritis (RA), accompanied by an acute-phase reactant elevation [105], and only 9% show seropositivity [106]. The seropositivity rate for rheumatoid factor(RF) in ICIs-IA patients is 5%, and the seropositivity rate for anti-citrullinated protein antibodies is 5.5% [107]. PET-CT and CT have shown high sensitivity and specificity in the diagnosis of synovitis in ICIs-IA patients [108]. Genetic evidence suggests that patients with at least one shared epitope (SE) allele associated with RA have an increased risk of developing ICI-IA, with the strongest associated risk allele being HLA DRB1*04:05 [109]. The absence of the PD1 gene or increased inhibition of PD-1 and PD-L1 increases the risk of various types of arthritis, systemic lupus erythematosus, and autoimmune encephalomyelitis [110, 111]. IA can mimic various inflammatory arthritis, including RA, reactive arthritis, osteoarthritis, and tenosynovitis. 11% of patients require DMARD therapy, and patients with RA-like symptoms are at higher risk of developing persistent arthritis. In tumor patients with coexisting osteoarthritis, ICI therapy may increase the risk of arthritis exacerbation, with 92% of patients experiencing grade 1–2 adverse events and 56% experiencing other types of irAEs. Low-dose corticosteroids can improve arthritis symptoms in these patients [112]. The severity of joint symptoms caused by ICIs varies, mostly ranging from mild to moderate. NSIADs or low-dose prednisone can improve symptoms without the need to stop ICI treatment. Increasing evidence suggests that ICIs-IA can persist long-term after discontinuing ICI treatment, becoming an important component of the spectrum of chronic irAEs diseases. Therefore, the management of ICIs-IA in cancer patients should be strengthened.

#### PMR and GCA

PMR is the second most common disease in Rh-irAEs, characterized by pain and stiffness in typical areas (shoulders, neck, hips, and back), which usually occurs 3 months after the use of ICIs and is often accompanied by other rheumatic irAEs such as IA [113]. PMR is more commonly seen with anti-PD1/PDL1 monotherapy, and the incidence of ICIs-related PMR is about 12.5% (17/136), with laboratory tests showing CRP levels higher than twice the upper limit of normal [2]. The incidence of PMR combined with GCA is relatively rare in patients with Rh-irAEs, but individual cases have been reported in patients undergoing temporal artery biopsy. Actively searching for evidence of temporal arteritis, such as headache, visual disturbances, and intermittent jaw movement disorders, is crucial for assisting in the diagnosis of PMR/GCA. The vast majority of ICIs-related PMR have a very good response to low to moderate dose steroid therapy, but if atypical manifestations or combined temporal arteritis occur, intensified treatment may be necessary.

#### Myositis

The incidence of PMR combined with GCA is relatively rare in patients with Rh-irAEs, but individual cases have been reported in patients undergoing temporal artery biopsy. Actively searching for evidence of temporal arteritis, such as headache, visual disturbances, and intermittent jaw movement disorders, is crucial for assisting in the diagnosis of PMR/GCA. The vast majority of ICIs-related PMR have a very good response to low to moderate dose steroid therapy, but if atypical manifestations or combined temporal arteritis occur, intensified treatment may be necessary [114]. In some cases, patients with ICIs-myositis may experience an elevation in creatine kinase (CK) levels of up to 70 times the normal value, accompanied by rhabdomyolysis [115]. Muscle pathology shows infiltration of cytotoxic T cells, multifocal necrotic muscle fibers, and endomysial inflammation, mainly composed of CD68 + PD-L1 + and CD8 + PD-1 + cells [116, 117]. The postmortem investigation into ICI-myositis has uncovered the existence of CD81 + T cells within tumors, cardiac tissue, and skeletal muscles, indicating a plausible correlation between muscle injury and the presence of cross-reactive T cells in both tumors and muscles [118]. This finding suggests a possible connection between muscle damage and the existence of T cells that demonstrate cross-reactivity within tumors and muscles, highlighting the need for further research in this area to elucidate the underlying mechanisms and inform potential therapeutic strategies for this condition. Myocarditis associated with ICI has a dismal prognosis, with a mortality rate of 51.5%, which is substantially higher compared to the 14.9% mortality rate in myositis patients without myocarditis. Early detection and timely intervention are imperative for this unique type of myositis, underscoring the need for vigilant monitoring of cardiac function, including a battery of diagnostic tests such as electrocardiography, echocardiography, and even cardiac magnetic resonance imaging (MRI) [119]. Generally, mild ICI-induced adverse events do not mandate discontinuation of ICI therapy, while severe or refractory cases require hospitalization and a broad array of therapeutic modalities, including corticosteroid pulse therapy, intravenous immunoglobulin, biologics, plasmapheresis, and immunosuppressive agents.

#### Sjögren syndrome

ICIs-induced Sjogren's syndrome(ICIs-SS) is primarily characterized by dry mouth and swollen salivary glands, with the majority of patients lacking antinuclear antibodies, anti-SSA/B antibodies, RF, and extractable nuclear antigens [120]. ICIs-SS is primarily caused by the combined therapy of CTLA4 and PD1 inhibitors (Ipilimumab and Nivolimumab), although there have been isolated cases resulting from the use of individual drugs or PDL1 blockers (Atezolimumab). Salivary gland biopsy is helpful for the diagnosis of ICIs-SS, which is characterized by marked lymphocyte infiltration (CD3 + and CD4 + T cells), epithelial damage, and B cell depletion. These histopathological features are consistent with those of primary Sjogren's syndrome (pSS) and support the notion that ICIs-SS and pSS common immunological mechanisms. In clinical practice, careful medical history and systematic evaluation, including diagnostic tests for oral and ocular dryness, serum autoantibodies, viral screening, and minor salivary gland biopsy, should be conducted for patients suspected of ICIs-SS to differentiate from other forms of sialadenitis. Mild symptoms can be controlled by local treatment and oral salivary gland function stimulators. When severe symptoms occur, ICI treatment should be temporarily discontinued, and oral corticosteroids may be considered.

#### Other diseases

The onset of vasculitis induced by immune checkpoint inhibitors (ICIs) often precedes that of IA and PMR. In contrast to vasculitis associated with other drugs, which mainly causes small-vessel vasculitis, ICI-related vasculitis primarily manifests as large-to-medium vessel vasculitis and neurovascular vasculitis. Daxini et al. [121] provided a comprehensive review of 20 previously reported cases of ICIs-related vasculitis. The study revealed that large vessel vasculitis, primarily GCA, accounted for the majority of cases (30%). This was followed by medium vessel vasculitis (25%), mainly peripheral neurovascular vasculitis, and single-organ vasculitis (20%), predominantly central nervous system vasculitis. Discontinuation of ICIs and high-dose corticosteroid immunosuppression can partially or completely alleviate the clinical manifestations in the majority of patients. Additionally, ICIs have been known to induce the occurrence of sarcoidosis, which manifests as benign skin nodules or systemic involvement of lymph nodes, lungs, or the nervous and ocular systems [122]. Due to the similarity in clinical presentation between sarcoidosis and ICIs-induced interstitial pneumonia, it can be challenging to differentiate the two in clinical practice. ICIs-related sarcoidosis typically presents with milder clinical symptoms and can be managed by temporarily discontinuing ICIs and administering corticosteroids.

## Discussion

PRSs can be difficult to distinguish from skin, muscle, and bone lesions caused by direct infiltration in classic autoimmune rheumatic diseases and tumors. Although the classification and diagnosis of rheumatic diseases often rely on clinical presentation, rheumatic-like manifestations do not necessarily equate to rheumatic diseases. When patients present with atypical rheumatic-like manifestations accompanied by unexplained fever, fatigue, weight loss, elevated tumor markers, unexplained masses, hepatosplenomegaly, lymphadenopathy, abnormal immunological markers, and poor response to conventional steroid and immunosuppressant therapies, malignancy screening should be considered as a priority. For cancer patients, a comprehensive assessment based on medical history, general physical condition, comorbid autoimmune diseases and their activity, laboratory and imaging examinations is necessary to determine whether they can continue to receive treatment with ICIs and AIs. Currently, the management of Rh-irAEs can be based on the grading of irAEs. Grade 1–2 irAEs are mild to moderate adverse reactions that do not require hospitalization. Grade 1 patients may receive appropriate analgesics (such as acetaminophen or NSAIDs), while Grade 2 patients may be treated with low-dose steroids for 4–6 weeks. Grade 3–4 irAEs are severe or life-threatening adverse reactions that require hospitalization for monitoring and treatment. In such cases, ICIs may need to be temporarily discontinued and high-dose steroids may be administered accordingly [123]. The diagnosis and management of rheumatic diseases associated with tumors pose considerable challenges. On one hand, this disease is infrequent and there is inadequate clinical research available. On the other hand, these diseases are often characterized by multifaceted conditions that necessitate a comprehensive approach, placing greater demands on clinical practitioners. In the future, it is necessary to strengthen the research on tumor-associated rheumatic diseases, explore their pathogenesis and treatment options, in order to improve our understanding and treatment level of this disease. At the same time, clinical physician training and skills enhancement should be strengthened to improve the diagnosis and treatment level of this disease and better serve the health of patients.

## Data Availability

Not applicable
